# Insulin-Activated Signaling Pathway and GLUT4 Membrane Translocation in hiPSC-Derived Cardiomyocytes

**DOI:** 10.3390/ijms25158197

**Published:** 2024-07-27

**Authors:** Giulia Querio, Susanna Antoniotti, Renzo Levi, Bernd K. Fleischmann, Maria Pia Gallo, Daniela Malan

**Affiliations:** 1 Department of Clinical and Biological Sciences, University of Turin, Regione Gonzole 10, 10043 Orbassano, Italy; giulia.querio@unito.it; 2 Department of Life Sciences and Systems Biology, University of Turin, Via Accademia Albertina 13, 10123 Turin, Italy; susanna.antoniotti@unito.it (S.A.); renzo.levi@unito.it (R.L.); 3 Institute of Physiology I, Medical Faculty, University of Bonn, 53127 Bonn, Germany; bernd.fleischmann@uni-bonn.de (B.K.F.); dmalan@uni-bonn.de (D.M.)

**Keywords:** hiPSC-CM, lactate medium, GLUT4, insulin, postnatal cardiac cell, cardiomyocyte, metabolism, cardiac maturation, plasma membrane translocation

## Abstract

Human induced pluripotent stem cell-derived cardiomyocytes (hiPSC-CM) are a cell model now widely used to investigate pathophysiological features of cardiac tissue. Given the invaluable contribution hiPSC-CM could make for studies on cardio-metabolic disorders by defining a postnatal metabolic phenotype, our work herein focused on monitoring the insulin response in CM derived from the hiPSC line UKBi015-B. Western blot analysis on total cell lysates obtained from hiPSC-CM showed increased phosphorylation of both AKT and AS160 following insulin treatment, but failed to highlight any changes in the expression dynamics of the glucose transporter GLUT4. By contrast, the Western blot analysis of membrane fractions, rather than total lysates, revealed insulin-induced plasma membrane translocation of GLUT4, which is known to also occur in postnatal CM. Thus, these findings suggest that hiPSC-derived CMs exhibit an insulin response reminiscent to that of adult CMs regarding intracellular signaling and GLUT4 translocation to the plasma membrane, representing a suitable cellular model in the cardio-metabolic research field. Moreover, our studies also demonstrate the relevance of analyzing membrane fractions rather than total lysates in order to monitor GLUT4 dynamics in response to metabolic regulators in hiPSC-CMs.

## 1. Introduction

Primary cultures of adult cardiomyocytes (CMs) are an important resource for studies of cardiac cell biology and physiology. However, despite their great potential, this cellular model presents some drawbacks [1,2]. Besides evident ethical and practical issues, a disadvantage in the use of isolated CMs is the reduced viability of these cells after tissue dissociation. Indeed, it is now well established that primary adult CMs in culture can maintain the typical rod-shaped appearance, and their physiology, for only a few days because of features of dedifferentiation, which significantly impacts potential experiments requiring longer culture times [1,2,3]. Therefore, there is a need for alternative approaches in our pursuit of studying and understanding cardiac diseases.

Human induced pluripotent stem cells (hiPSCs), with their capacity to differentiate in all three embryonic germ layers, figure as a promising tool to obtain postnatal cardiac cells [4]. Indeed, hiPSCs can be differentiated into mesoderm progenitor cells and, through widely accepted protocols, develop towards the cardiac lineage generating human induced pluripotent stem cell-derived cardiomyocytes (hiPSC-CMs) [4,5,6]. HiPSC-CMs bear great potential, as these are human cells (reprogrammed from somatic patient cells). Therefore, they represent the best cellular model for studying human cardiac physiology and a significant milestone towards a more individual-based approach. However, many studies highlight that hiPSC-CMs do not recapitulate adult CM features, as their morphological, electrophysiological, and metabolic properties [7,8,9] are much less mature. New culture strategies are therefore being studied to obtain CMs that, from a morphological, functional, and metabolic point of view, are more similar to terminally differentiated CMs. Such approaches comprise longer culture times [10,11], the addition of culture medium components [12,13,14] for 2D models, or 3D models [7,15,16] possibly yielding a more mature CM phenotype.

The maturation of hiPSC-CM has already been investigated in detail, as pointed out above, in morphological and electrophysiological terms [5,17,18], but less is known regarding their metabolic maturation [13,19]. During their physiological maturation, CMs undergo a metabolic switch [7,8,20,21], as they transit from a predominantly carbohydrate metabolism in the fetal period to a phase in which carbohydrates and lactate constitute the main energy substrates in the perinatal time, up to the postnatal/adult period. In this last stage, CMs acquire metabolic flexibility, as they can shift from fatty acid as the main substrate to glucose, depending on the nutritional status and physical activity [22,23,24]. This metabolic flexibility is strictly related to a proper response to insulin; in particular, the activation of the insulin receptor starts the intracellular cascade that ends with the translocation of the GLUT4 transporter from intracellular vesicles to the plasma membrane, accounting for the beneficial shift in cardiac energy substrate towards glucose oxidation [25]. The insulin response is therefore of fundamental importance in the regulation of the metabolic switch that characterizes healthy adult cardiomyocytes. This ability may fail in certain pathological conditions, such as insulin resistance and diabetes, characterized by the absence of response to insulin stimulus and a metabolic rigidity that is often associated with cardiac dysfunction [26]. The availability of a cellular model that presents the metabolic characteristics of an adult cardiomyocyte represents a point of fundamental importance for studying the various pathological and metabolic conditions associated with insulin resistance and diabetes. Thus, the insulin-activated GLUT4 translocation to the plasma membrane is pivotal for the characterization of the metabolic maturity of the hiPSC-CM model.

Building on the insights from Keiichi Fukuda’s research and in alignment with the findings of Kadari et al., we took advantage of an incubation period using a lactate-containing medium as the exclusive energy source after differentiating cardiomyocytes (CMs). This approach, as demonstrated by these authors, allowed us to employ a metabolic-based selection method, resulting in the purification of a greater quantity of cells [14,27]. We therefore focused on the characterization of the insulin response in the hiPSC-CM. To this aim, we investigated whether in differentiated hiPSC-CM cells, short-term stimulation (30 min) with insulin was able to activate key elements of the insulin receptor signaling pathway involved in GLUT4 translocation, namely AKT and AS160, and whether it could induce an increase in GLUT4 in the membrane fraction, as reported for adult CMs [28]. The insulin-mediated effect on GLUT4 was verified with Western blot measurements both on total cell lysates and on membrane fractions.

## 2. Results

### 2.1. HiPSC-CMs Express Key Components of the Insulin-Activated Pathway

**Figure 1:**
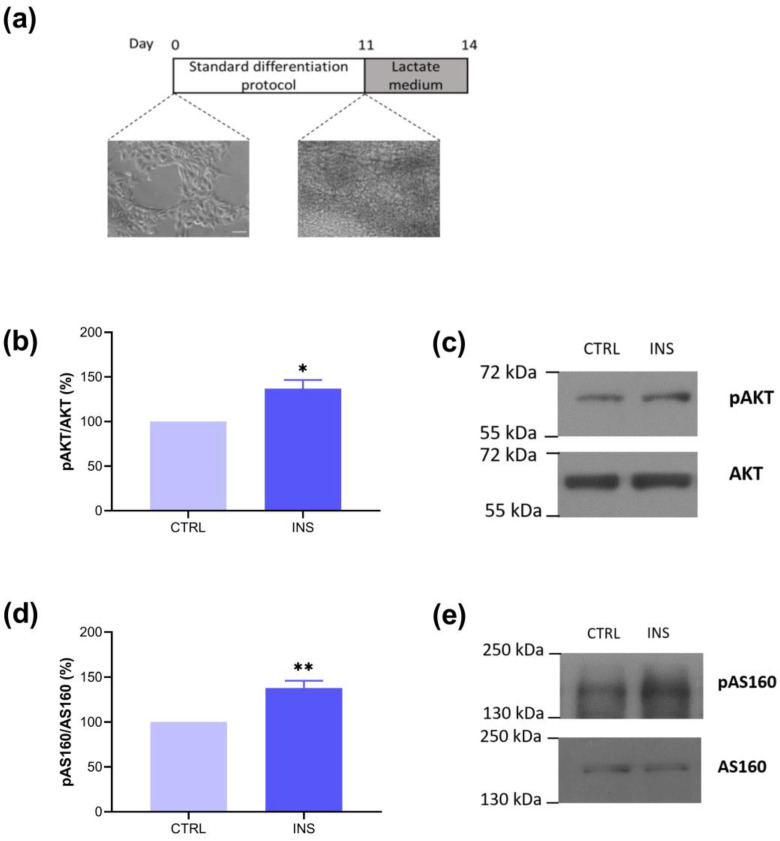
Differentiated hiPSC-CMs show core elements of the insulin signaling pathway. (**a**) Schematic representation of the differentiation protocol. Cells were differentiated with standard protocol, and then, from days 11 to 14, incubated with lactate medium before the experiments. Bright field acquisitions of undifferentiated (left) and differentiated (right) cells (20×, scale bar 5 µm). Bar graph and representative blots of lactate-purified hiPSC-CMs treated or not with insulin (INS, 100 nM, 30 min) showing (**b**,**c**) pAKT/AKT (CTRL: 100.00; INS: 136.86 ± 9.80) and (**d**,**e**) pAS160/AS160 (CTRL: 100.00; INS: 137.93 ± 8.12). n = 3 independent experiments, * *p* < 0.05; ** *p* < 0.01.

UKBi015-B derived from healthy donors were differentiated into CMs (from here and throughout the text, referred to as hiPSC-CMs) according to the differentiation media instructions (see Section 4). Once the cells began to contract spontaneously (around day 8), they were incubated from days 11 to 14 with a medium containing lactate as the only energy substrate (Figure 1a), as this step has already been established to enrich the number of CMs and make more comparable the different preparations [14,27].

First, experiments were addressed to assess if our model of hiPSC-CMs expresses key components of the insulin signaling pathway, such as phosphorylated AKT (Ser473) (pAKT) and phosphorylated AS160 (Thr642) (pAS160). In particular, the GTPase activating protein (GAP) AS160 represents a pivotal substrate of AKT, as its phosphorylation results in the inhibition of the GTPase activity on the small G-protein of the Rab family and in the consequent ignition of vesicular traffic and fusion with the plasma membrane [29]. Figure 1b,d show that after insulin stimulation (INS, 100 nM, 30 min), hiPSC-CMs express higher levels of both pAKT and pAS160, indicating the activation of the insulin signaling pathway.

### 2.2. Insulin-Stimulated GLUT4 Membrane Translocation Is Visible Only on Membrane Lysates

**Figure 2:**
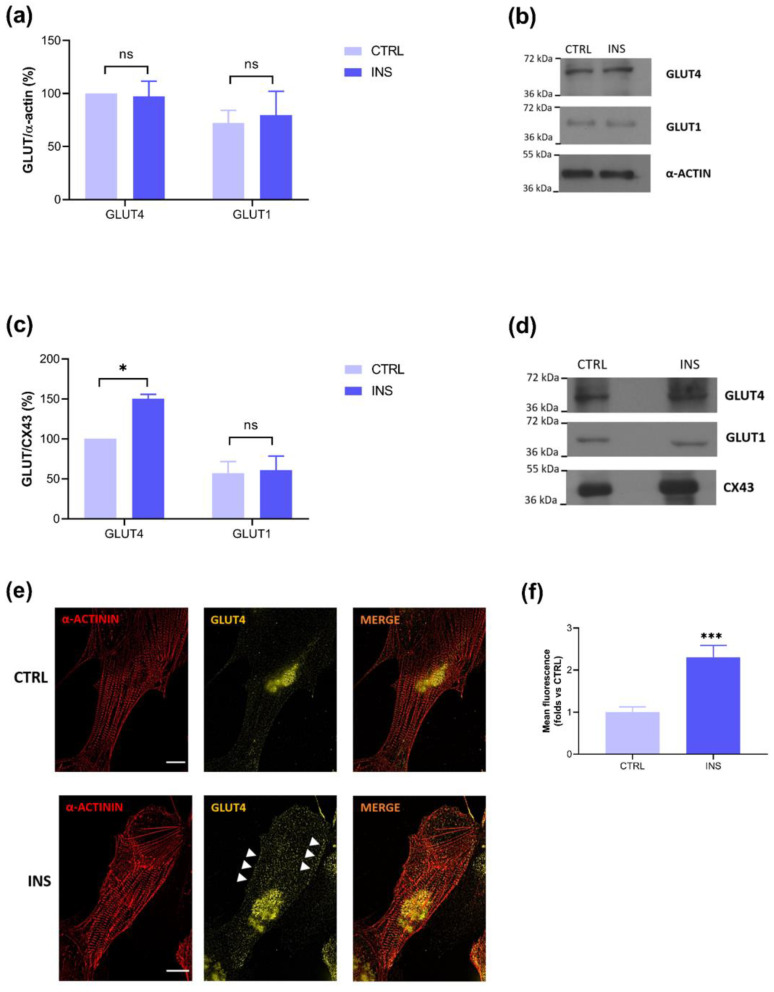
Lactate-purified hiPSC-CMs show significant GLUT4 membrane translocation after insulin stimulation. Bar graph and representative blots of (**a**,**b**) total lysates and (**c**,**d**) membrane fractions showing higher GLUT4 levels in membrane fractions of insulin-treated cells. GLUT1 shows no variations after insulin stimulation. (Total lysates: CTRL-GLUT4: 100.00; INS-GLUT4: 97.33 ± 14.33, CTRL-GLUT1: 72.18 ± 12.04; INS-GLUT1: 79.51 ± 22.60; Membrane fractions: CTRL-GLUT4: 100.00; INS-GLUT4: 150.34 ± 5.49, CTRL-GLUT1: 57.26 ± 14.42; INS-GLUT1: 61.04 ± 17.60). (**e**) Representative immunostaining showing GLUT4 membrane translocation in insulin-treated cells (white arrows) (40×, scale bar 10 µm), and (**f**) the bar graph summarizing the immunofluorescence experiments analysis in 34 α-actinin-positive cells for both conditions (CTRL: 1.00 ± 0.13; INS: 2.31 ± 0.28). n = 3 independent experiments, * *p* < 0.05; *** *p* < 0.001.

To assess if, after the activation of the insulin signaling core elements, pAKT and pAS160, a membrane translocation of the glucose transporter GLUT4 occurs, like in adult CMs [25,28], Western blots of total and membrane lysates were performed. As Figure 2a shows, the effect of insulin on GLUT4 translocation was not visible on total lysates, and these data were confirmed monitoring the non-insulin dependent glucose transporter GLUT1, which, as expected, showed no changes after INS treatment. Conversely, a significant difference in GLUT4 expression following insulin stimulation was detected on membrane lysates, whereas GLUT1 expression, here monitored as a non-insulin dependent transporter, remained constant following INS treatment (Figure 2c). This finding was further confirmed by immunocytochemistry experiments (Figure 2e,f). These data show the importance and better efficacy of using membrane vs. total lysates analysis for the assessment of GLUT4 trafficking.

## 3. Discussion

This short study aims to demonstrate that hiPSC-derived CMs (UKBi015-B line) respond to insulin stimulation in terms of the phosphorylation of AKT and its substrate AS160, which are known to be directly involved in GLUT4-containing vesicle trafficking. Moreover, we also show that this pathway leads to GLUT4 translocation to the plasma membrane, as highlighted on isolated membrane fractions rather than total cell lysates.

Currently, it is widely accepted that the physiological metabolic maturation that characterizes cardiomyocytes starts in the fetal/perinatal period with an initial utilization of glucose and lactate as the main energy sources. However, in the adult phase, there is a notable shift towards the utilization of fatty acids, covering approximately 70% of the cardiac energy requirements [20]. The use of different substrates during metabolic maturation is accompanied by a change in the expression of membrane transporters. Indeed, the most strongly expressed glucose transporter in postnatal and adult CMs is GLUT4, an insulin-dependent transporter whose membrane translocation is achieved by insulin-activated intracellular signaling [25]. All these aspects that characterize the maturation of cardiomyocytes towards an adult stage are inevitably marked by specific responses to hormonal stimulation. In particular, the response to insulin is the one that characterizes the metabolic flexibility of adult cardiomyocytes. In fact, insulin binding to its receptor activates an intracellular cascade that ends with the membrane translocation of the GLUT4 transporters, facilitating glucose uptake. This tightly controlled metabolic response allows cardiomyocytes to use different metabolic substrates as needed. An alteration of the insulin response, as seen in insulin resistance or diabetes, can cause the loss of the metabolic flexibility that characterizes cardiac cells, potentially causing the development of diabetic cardiomyopathy [26].

The availability of a cellular model with the metabolic characteristics of an adult cardiomyocyte represents a point of fundamental importance for studying the pathophysiological variations that occur in the cardiac tissue.

Our first experiments, therefore, explored the expression of the main components of one of the insulin-activated glucose uptake pathways, pAKT and pAS160, in our lactate- purified hiPSC-CMs model. Indeed, insulin binding to its receptor induces the activation of phosphoinositide 3-kinase isoform α (PI3Kα) that, through the mammalian target of rapamycin complex 2 (mTORC2), increases AKT phosphorylation at Ser473 (pAKT). Phosphorylated AKT enhances the AKT substrate of 160 kDa phosphorylation (pAS160). Phosphorylated AS160 reduces its GTPase-activating function and activates Rab proteins, thus enabling GLUT4-containing vesicles trafficking towards the plasma membrane. As Figure 1 shows, both pAKT and pAS160 were expressed after insulin treatment in our model, suggesting the activation of the pathway that characterizes glucose uptake after insulin stimulation in postnatal and adult CMs [28,29,30]. These results are in accordance with those from Bowman and collaborators, who showed an increase in the pAKT/Total AKT ratio in their model of hiPSC-CMs after insulin stimulation [31]. Furthermore, Jung and collaborators showed an increased expression of the pAKT/Total AKT ratio, directly proportional to differentiation times, reporting the maximum phosphorylation of AKT after 90 days [32].

As one of the responses triggered by the insulin pathway is the translocation of GLUT4-containing vesicles from the cytoplasmic to the membrane compartment to promote glucose uptake, our attention turned to the evaluation of the expression of this transporter in our model of hiPSC-CMs and its membrane translocation following insulin stimulation. We therefore conducted Western blot on the total lysates to evaluate the GLUT4 expression in lactate-purified hiPSC-CMs both in the control condition and after insulin stimulation. The results obtained showed the expression of the glucose transporter, but no differences between the control and insulin-treated cells were detected (Figure 2a). These last findings were probably due to the large leakage of membrane proteins in the totality of cell proteins. In fact, the evaluation on total lysates did not allow us to distinguish the various cellular compartments, while the insulin effect on GLUT4 is focused on a plasma membrane dynamic. This result contrasts with that of Bowman and coworkers, who did not detect GLUT4 expression, or subsequent glucose uptake after insulin treatment, in their cell model [31]. Possible differences may be related to the origin of the studied hiPSC-CMs, which in the case of Bowman and coworkers, were commercially obtained, whereas in our study, they were differentiated from reprogrammed somatic patient-derived cells. Thus, we can state that our hiPSC-CM model differs from other commercial ones, showing features that most closely approximate that of a postnatal phenotype capable of expressing GLUT4.

These first promising results showing GLUT4 expression in our model of hiPSC-CMs motivated us to deepen the evaluation of its insulin-dependent translocation on membrane fractions.

To do this, we isolated membrane fractions through a sucrose buffer and high-*g* force centrifugation protocol [33]. In this more specific experimental setting, Western blot analysis showed significantly higher GLUT4 expression in insulin-treated cells (Figure 2c), and this result was supported by an immunofluorescence assay (Figure 2e,f), providing robust evidence of the insulin-dependent translocation of GLUT4 to the membrane fractions. Furthermore, since our model also shows the expression of GLUT1, this leads us to underline that, with the differentiation protocol we used, the metabolic profile that is reached still resembles that of a postnatal stage rather than definitively adult. Moreover, GLUT1, being a non-insulin-dependent transporter, served as a control for the detection of GLUT4 membrane translocation following insulin stimulation, a response that could only be visualized by isolating the membrane fractions (Figure 2).

A final consideration in our work relates to the choice of housekeeping controls for Western blot analysis: α-sarcomeric actin for total lysates and connexin 43 (CX43) for membrane lysates. As both proteins were expressed in our lysates, one can speculate that the assays were performed on more mature-like CMs because α-sarcomeric actin has been identified as the primary actin isoform in more differentiated CMs [34], and CX43 is the principal gap junction connexin in healthy working myocardium [35]. This hypothesis is partly reinforced by the results of Kadari et al., from which the lactate incubation protocol of the present work was taken [14].

Inevitably, this study presents some limitations, principally linked to the brief maturation time used for the differentiation of the hiPSC-CMs. Indeed, earlier work pointed out that time-dependent maturation could be a useful tool to obtain more adult-like cells [10,19]. On the other hand, our approach also has advantages, namely the use of a highly reproducible, standardized differentiation protocol and also the isolation of membrane fractions, which offers a suitable option, with respect to total lysates, for Western blots aimed to highlight membrane protein dynamics. Our experiments were performed on the UKBi015-B line, which demonstrates proven pluripotency as indicated in the characterization of the line in the hiPSC scorecard (https://hpscreg.eu/cell-line/UKBi015-B, accessed on 1 January 2020). Although the data obtained could be strengthened with a parallel study with other hiPSC lines, the cardiac differentiation of this line demonstrates the profound utility of hiPSC-CMs and promises a broader understanding of cardiac development and function. What strengthens our results is the similarity to the insulin response in postnatal CMs and the possibility of using our model to modulate metabolic disorders such as diabetic cardiomyopathy [36].

## 4. Materials and Methods

### 4.1. Reagents

Undifferentiated hiPSCs were maintained in StemMACS™ iPS-Brew XF (#130-104-368, Miltenyi Biotec, Bergisch Gladbach, Germany). RevitaCell™ Supplement 100× (#A2644501, Life Technologies Corporation, Carlsbad, CA, USA), diluted 1:100, was added to the culture medium at each cell passage. The detachment of undifferentiated cells was performed with EDTA in DPBS 0.5 mM (Sigma-Aldrich, Merck Group, Darmstadt, Germany).

Cardiac differentiation of hiPSC was performed according to the StemMACS^TM^ CardioDiff Kit XF, human (#130-125-289, Miltenyi Biotec). After differentiation, cells were maintained from day 11 to day 14 in lactate medium composed of Dulbecco’s Modified Eagle Medium (DMEM, #11966-025, Gibco, Thermo Fisher Scientific, Waltham, MA, USA), without glucose and sodium pyruvate, and with L-glutamine, with 1% nonessential amino acids (#11140-35, ThermoFisher Scientific), 1% penicillin/streptomycin (#15140-122, ThermoFisher Scientific), 1% transferrin selenium (#S5261, #T8158, Sigma-Aldrich), 0.2% β-mercaptoethanol (#31350-010, Thermo Fisher Scientific), and lactate (#71718, Sigma-Aldrich) added to the final concentration of 4 mM. Matrigel^®^ Matrix (#356234, Corning, NY, USA) at the dilution 1:100 in KnockOut^TM^ DMEM (#10829018, ThermoFisher Scientific) was used to coat plates for undifferentiated and differentiated cells. Human insulin (#I2643, Sigma-Aldrich) was used for cell treatments.

### 4.2. Cell Line

Undifferentiated hiPSC line iLB-C16m-s16 (UKBi015-B) (https://hpscreg.eu/cell-line/UKBi015-B, accessed on 1 January 2020) was kindly provided by Dr. Michael Peitz, Cell Programming Core Facility, University of Bonn Medical Faculty, and Prof. Dr. Oliver Brüstle, Institute of Reconstructive Neurobiology, University Hospital Bonn.

Cells were maintained in StemMACS^TM^ iPS-Brew XF added with RevitaCell™ Supplement 100×, diluted 1:100, on the first day of plating. Cells were incubated at 37 °C in a humidified atmosphere containing 5% CO_2_ during all experiments. HiPSCs were used from passage 2 to 10.

### 4.3. Differentiation Protocol and Treatment

The cardiac differentiation of the hiPSCs was induced with StemMACS^TM^ CardioDiff Kit XF, human. Cells were plated on Matrigel-coated plastic petri dishes with 22.1 cm^2^ of growth area according to the manufacturer’s instructions. Daily medium replacement was performed as recommended by the protocol, and around day 8 of differentiation, cells started contracting. From days 11 to 14 of differentiation, cells were incubated with lactate medium. At day 14 of differentiation, part of the plated cells were treated with insulin (INS) 100 nM for 30 min and part were left in the same medium (CTRL) before the experiments.

### 4.4. Total Lysates

Differentiated hiPSC-CMs maintained in lactate medium were treated with insulin 100 nM for 30 min to monitor any variation in pAKT, pAS160, and GLUT4, in total lysates.

After the treatment, cells were lysed on ice with Pierce^TM^ RIPA buffer (#89900, Thermo Fisher Scientific) containing protease inhibitor cocktail (cOmplete^TM^, Mini, EDTA-free, #04693159001, Roche, Mannheim, Germany) and 1 mM PMSF, forced through a 1 mL syringe needle several times, centrifuged at 10,000 rpm for 5 min at 4 °C, and stored at −80 °C.

### 4.5. Isolation of Membrane Lysates

The isolation of membrane fractions was performed according to Antoniotti et al. [33] with some modifications. Briefly, cell lysates were obtained with lysis buffer composed of Tris 25 mM (pH 7.4), 0.3 M sucrose, 1 mM PMSF, and 2 µg/mL aprotinin. Lysates were collected on ice, homogenized through a syringe needle, and centrifuged at 8000× *g* for 15 min at 4 °C. The resulting supernatant was then centrifuged at 40,000× *g* for 1 h 30 min at 4 °C, and the supernatant containing the cytoplasmic fraction was isolated from the pellet, which contained the membrane fraction that was suspended in 200 µL of lysis buffer and stored at −80 °C.

### 4.6. Immunoblotting

After INS stimulation, protein expression was evaluated with Western blotting both in total lysates and membrane fractions. Total lysate (20 µg per lane) and membrane fractions (30 µL per lane) were run on a 12% SDS-PAGE gel, transferred to a polyvinylidene fluoride membrane (PVDF), and blocked in 5% non-fat dry milk in TBST 1× (Tris-HCl 10 mM, pH 7.5, NaCl 0.1 M, Tween 20 0.1%) at 37 °C for 1 h. PVDF was incubated overnight at 4 °C with primary antibodies (monoclonal AKT (pan), #C67E7, Cell Signaling Technology, Danvers, MA, USA, dilution 1:100; monoclonal phospho-AKT(Ser473), #D9E, Cell Signaling Technology, dilution 1:1000; monoclonal AS160, #C69A7, Cell Signaling Technology, dilution 1:1000; polyclonal phospho-AS160(Thr642), #44-1071G, Thermo Fisher Scientific, dilution 1:1000; polyclonal anti-GLUT4, #PA5-23052, Thermo Fisher Scientific, dilution 1:500; monoclonal anti-GLUT1, #MABS132, Sigma-Aldrich, dilution 1:400; polyclonal anti-connexin-43, #C6219, Sigma-Aldrich, dilution 1:4000; monoclonal anti-α-sarcomeric actin, #A2172, Sigma-Aldrich, dilution 1:5000). Membranes were then washed three times with TBST 1× and incubated with horseradish peroxidase-conjugated secondary antibody for 1 h at room temperature (anti-rabbit, #SA00001-2, Proteintech Group, Rosemont, IL, USA, dilution 1:10,000, and anti-mouse, #31430, Thermo Fisher Scientific, dilution 1:20,000), and then washed again three times with TBST 1×. Protein bands were localized by chemiluminescence with Western Lighting Plus-ECL (Perkin Elmer, Waltham, MA, USA). Protein levels were determined with the software ImageJ (Rasband, W. S., ImageJ, U. S. National Institutes of Health, Bethesda, MD, USA; https://imagej.nih.gov/ij/. Version: 2.9.0/1.53t, 2010–2024) and expressed as the mean percentage of pAKT/Total AKT normalized with corresponding α-actin, pAS160/AS160 normalized with corresponding α-actin, GLUT4/α-actin, GLUT1/α-actin, or GLUT4/CX43, and GLUT1/CX43 ratios of three independent experiments ± s.e.m.

### 4.7. Immunofluorescence

HiPSC-CMs were dissociated after 14 days and were fixed with 4% formaldehyde for 20 min and later stained with, respectively, primary antibodies against α-actinin (#A7732, Sigma Aldrich, dilution 1:400) and GLUT4 (#PA5-23052, Thermo Fisher Scientific, dilution 1:100) diluted in PBS with 0.2% Triton X-100 and 5% donkey serum for 24 h at 4 °C. Alexa-Fluor 555 anti-rabbit IgG or Alexa Fluor 647 goat anti-mouse IgG1 (Thermo Fisher Scientific) secondary antibodies were applied at a dilution of 1:400 for one hour at room temperature. The immunostainings were documented with a Nikon microscope (eclipse Ti2, Nikon, Minato, Tokyo, Japan) using the deconvolution function. GLUT4 staining measurements of both the cell periphery and cell interior were performed with the ImageJ software (Rasband, W. S., ImageJ, U. S. National Institutes of Health, Bethesda, MD, USA; https://imagej.nih.gov/ij/. Version: 2.9.0/1.53t, 2010–2024). Briefly, for each α-actinin-positive cell, the contour was limited, and the region of interest (ROI) as a band of 8 pixels towards the inside of the cell was isolated. The ROI’s fluorescence intensities of each selected cell were evaluated and expressed as folds towards the CTRL ± s.e.m.

### 4.8. Statistical Analysis

Data are presented as mean ± s.e.m. and were analyzed with GraphPad Prism 8.0.1 software using an unpaired *t* test or 2-way ANOVA followed by Bonferroni’s post hoc test. Differences with *p* < 0.05 were considered statistically significant.

## Data Availability

The data presented in this study are available on request from the corresponding author. The data are not publicly available due to their repositories at the University of Turin.
